# Unveiling immune mechanisms and potential biomarkers in intervertebral disc degeneration through integrated analysis

**DOI:** 10.1590/1414-431X2025e14553

**Published:** 2025-06-20

**Authors:** Xuehu Xie, Guoqiang Zhang, Ning Liu

**Affiliations:** 1 Department of Orthopedics, Beijing Friendship Hospital, Capital Medical University, Xicheng District, Beijing, China

**Keywords:** Intervertebral disc degeneration, Immune, Immune-related differentially expressed mRNAs, Functional enrichment, ceRNA network

## Abstract

Immune regulation plays an important role in the pathogenesis of intervertebral disc degeneration (IDD). However, the mechanism of immune regulation in IDD is still unclear. All IDD data were downloaded from a public database. The differentially expressed (DE) immune-related genes in IDD were identified by the limma package in R. Functional enrichment analyses were performed to explore potential immune-related biological pathways in IDD. We also identified differentially expressed microRNAs (miRNAs) and long non-coding RNAs (lncRNAs) and constructed an mRNA-miRNA-lncRNA network. ROC analysis was performed to reveal potential diagnostic biomarkers for IDD. To understand the potential role of immune cells in IDD, xCell and Pearson correlation analyses were performed. Finally, expression validation was performed using real-time PCR. C5AR2, NFATC2, FCGR3A, hsa-miR-302d-3p, and MIR17HG were identified in IDD. ROC analysis results suggested that C5AR2 had good diagnostic accuracy, and FCGR3A and NFATC2 had sufficient diagnostic accuracy, which implied that they may be potential diagnostic markers of IDD. We also found that a large number of immune-related signaling pathways, such as cytokine-cytokine receptor interaction, chemokine signaling pathway, toll-like receptor signaling pathway, and Nod-like receptor signaling pathway, were significantly enriched. C5AR2, hsa-miR-302d-3p, and MIR17HG were significantly correlated with multiple immune cell types, such as cDC, CD8+ Tem, macrophage M1, neutrophils, and plasma cells. The C5AR2-hsa-miR-302d-3p-MIR17HG axis may play a role in immune regulation by regulating the infiltration level of related immune cells in the IDD microenvironment. The identification of key immune-related molecules, cells, and signaling pathways in IDD is of great significance to reveal the pathogenesis of IDD.

## Introduction

The intervertebral disc is embedded between the vertebrae to provide flexibility for the spine. It is composed of the nucleus pulposus, the annulus fibrosus, and the cartilage endplate. Compared with the non-degenerated intervertebral disc, the height of the degenerated disc is significantly reduced, the fibrous nucleus pulposus is dehydrated, the fibrous annulus bends inwards and outwards, the endplate is extensively damaged, and the subchondral bone is sclerotic (1). Intervertebral disc degeneration (IDD) is the leading cause of lower back pain, which can lead to severe disability (2). Low back pain is a common disease worldwide, which poses a heavy burden on public health and economy. Currently, the commonly used clinical treatments for IDD include conservative treatment (physical therapy, drug therapy) and surgical treatment (intervertebral disc fusion, total disc replacement) (3). However, these treatments can only temporarily relieve the pain symptoms and cannot solve the fundamental problem of IDD. The continuous research into biotherapy may provide a new direction for the treatment of IDD. However, due to the unclear molecular and pathological mechanisms of IDD, it is difficult to advocate biotherapy in clinical medicine. Therefore, it is necessary to further investigate the underlying mechanisms of IDD to help in the treatment, diagnosis, and management of IDD.

More and more studies have shown that immune regulation plays an important role in the pathogenesis of IDD. Although the nucleus pulposus is an immune-privileged organ, destruction of the physical barrier between the nucleus pulposus and the systemic circulation or innervation and vascularization of the degenerated nucleus pulposus result in exposure of the nucleus pulposus to the immune system as a foreign antigen on the one hand, and compression of nerve roots or dorsal root ganglia on the other hand, which will cause immune responses induced by immune cells and their mediators (4). Inflammation plays a crucial role in the progression of IDD in humans, and macrophages are considered to be key immune cells in this process (5). IDD also has different immune patterns (6). Immune-related genes undoubtedly affect immune processes, which can be reflected by changes in immune cell composition. Immune-related genes may play an important role in the pathogenesis of IDD by regulating immune cells and immune-related pathways. Although the above studies have shown that immune regulation plays an important role in the progression of IDD, the specific immune mechanism in IDD is not clear due to the complexity of the pathological mechanism of IDD. Therefore, the continuous study of immune cells and signaling pathways that may play a role in IDD can help reveal the pathogenesis of the disease, which is of great significance for the treatment and management of patients.

xCell is a new method combining the advantages of gene set enrichment with the deconvolution method, which can convert the gene expression profile into the enrichment scores of 64 immune and stroma cell types in the sample. Currently, xCell is often used to evaluate the degree of infiltration of immune cells in the microenvironment.

In this study, we aimed to understand the molecular and immune regulation mechanisms of IDD and hope to contribute to the clinical diagnosis, treatment, and management of IDD.

## Material and Methods

### Data sources of IDD

IDD-related datasets were retrieved from the Gene Expression Omnibus (GEO) database. Subsequently, the dataset containing genome-wide messenger ribonucleic acid/microRNA/long non-coding RNA (mRNA/miRNA/lncRNA) transcriptome data was selected. Furthermore, mRNA/miRNA/lncRNA transcriptome data were obtained from the nucleus pulposus of IDD and control groups. Standardized or raw datasets that meet the above requirements were considered for this study. Finally, the GSE167199 dataset was obtained for this study. The nucleus pulposus tissue samples were obtained from IDD patients and tissues from spinal cord injury were obtained as controls (7). Both IDD and control groups contained 3 sequencing samples. The degree of disc degeneration was assessed using magnetic resonance imaging scans in accordance with the Pfirrmann grading classification.

### Identification of immune-related differentially expressed mRNAs (DEmRNAs) in IDD

The GSE167199 mRNA dataset was downloaded from the GEO database. The mRNA expression data in the dataset was logarithmically processed. Subsequently, the limma package in R software was used for differential analysis. The screening criteria of DEmRNAs were |logfold change| (|logFC|)>1 and false discovery rate (FDR)<0.05. Immune-related genes were downloaded from the immport database (https://www.immport.org/shared/home). Then, immune-related genes and DEmRNAs were intersected to obtain immune-related DEmRNAs of IDD.

### Functional enrichment analysis of immune-related DEmRNAs

The David database (https://david.ncifcrf.gov/tools.jsp) was used for Gene Ontology (GO) and Kyoto Encyclopedia of Genes and Genomes (KEGG) functional enrichment analysis of immune-related DEmRNAs. The screening criteria was P<0.05. GO functional enrichment aspects include biological process (BP), cellular component (CC), and molecular function (MF). In addition, the enrichment analysis of Gene Set Enrichment Analysis (GSEA) was also carried out using GSEA software tools (http://www.gsea-msigdb.org/gsea/index.jsp).

### Identification of differentially expressed miRNAs (DEmiRNAs) and differentially expressed lncRNAs (DElncRNAs)

The GSE167199 miRNA dataset and GSE167199 lncRNA dataset were downloaded from the GEO database. The miRNA and lncRNA expression data in the dataset was logarithmically processed. Subsequently, the limma package in R software was used for differential analysis. The screening criteria of DEmiRNAs and DElncRNAs were |logFC|>1 and FDR<0.05.

### Construction of the lncRNA-miRNA-mRNA (ceRNA) network

The StarBase (https://starbase.sysu.edu.cn/) software was used to predict the miRNAs targeted to DElncRNAs. The predicted miRNAs were intersected with DEmiRNAs to obtain negatively regulated lncRNA-miRNA targeting relationship pairs. The miRWalk (http://mirwalk.umm.uni-heidelberg.de/interactions/) software was used to predict the targeted mRNAs of DEmiRNAs. The predicted targeted mRNAs and immune-related DEmRNAs were intersected to obtain the negatively regulated miRNA-mRNA targeting relationship pairs. Subsequently, the lncRNA-miRNA targeting relationship pairs and miRNA-mRNA targeting relationship pairs were fused to construct a ceRNA network.

### Analysis of cell infiltration in the IDD microenvironment

The xCell (https://xcell.ucsf.edu/) software is based on the ssGSEA method to calculate the xCell score of 64 immune cells and stromal cells in samples. XCell score was sorted into the cell infiltration matrix, and cell types with significant differences between IDD and control groups were obtained by difference analysis. Pearson correlation analysis was used to analyze the correlation between differential infiltration cell types and complement C5a receptor 2 (C5AR2), nuclear factor of activated T cells 2 (NFATC2), Fc gamma receptor IIIa (FCGR3A), hsa-miR-302d-3p, and miR-17-92a-1 cluster host gene (MIR17HG). The cut-off value for significance was P<0.05.

### Receiver operator characteristic (ROC) curve analysis

The GSE150408 dataset included 17 IDD and 17 control samples. GSE150408 data were from 17 patients with sciatica confirmed by magnetic resonance imaging and 17 healthy volunteers without clinical evidence of low back pain or sciatica (8). The specimen was blood from the participant's left median elbow vein. ROC curve analysis and electronic expression verification were performed based on the GSE150408 dataset. ROC analysis was performed using the pROC package (http://web.expasy.org/pROC/) in R software to assess the potential diagnostic value of DEmRNAs. In ROC curve analysis, the area under the curve (AUC) is often used to evaluate diagnostic accuracy. 0.6<AUC<0.7 indicates sufficient diagnostic accuracy, and AUC>0.7 indicates good diagnostic accuracy.

### Validation of real-time PCR

The inclusion criteria for IDD patients were: 1) meeting the clinical diagnostic criteria for IDD; 2) first onset of the disease; 3) being older than 18 years; 4) having complete clinical data, including gender, age, height, weight, etc. The exclusion criteria for IDD patients were: 1) multi-segmental lumbar lesions and severe dysfunction of other systems; 2) bilateral lower limbs sensorimotor abnormalities, abnormal urination and defecation function, lumbar scoliosis or kyphosis, deformity in other parts of the spine, thoracic tumors and thoracic tuberculosis, and other cervical thoracolumbar diseases; 3) rheumatism, mandatory spondylitis and other autoimmune diseases, lumbar infection, trauma, surgery history; 4) recurrence; 5) incomplete clinical data or unwilling to participate in the study. The control group was composed of individuals who matched the disease group in gender and age, did not suffer from intervertebral disc disease, and had complete clinical data.

In total, 27 blood samples (12 IDD and 15 control) were collected from the population examined and treated in our hospital. Detailed clinical information is shown in Supplementary Table S1. This study was approved by the Bioethics Committee of Beijing Friendship Hospital, Capital Medical University (2023-P2-024-01). Written informed consent was obtained from all patients. RNAliquid ultrafast whole blood (liquid sample) total RNA extraction kit (Beijing Huitian Dongfang Technology Co., LTD, China) was used for total RNA extraction. FastQuant cDNA first strand synthesis kit (TIANGEN, China) was used for mRNA reverse transcription. miRNA first strand cDNA synthesis kit (tail adding method) (Sangon Biotech, China) was used for miRNA reverse transcription. SuperReal PreMix Plus (SYBR Green) (TIANGEN) and miRNA fluorescent quantitative PCR kit (dye method) (Sangon Biotech) were used for real-time PCR validation of mRNA and miRNA. ABI 7300 fluorescence quantitative PCR instrument (Applied Biosystems, USA) was used to perform real-time PCR. Each experiment was repeated three times. GAPDH, ACTB, and hsa-U6 were used as internal references. Hsa-U6 primers and hsa-miR-302d-3p reverse primer were obtained from the miRNA first strand cDNA synthesis kit (Sangon Biotech). The 2^-△△CT^ method was used for relative quantitative analysis.

### Statistical analysis

All statistical analyses in this study were completed in R software. The limma package in R software was used to identify DEmRNAs, DEmiRNAs, and DElncRNAs, and the screening criteria were |logFC|>1 and FDR<0.05. The *t*-test was used to analyze the difference of xCell score between IDD and normal control groups. P<0.05 was considered statistically significant.

## Results

### Identification and functional enrichment analysis of immune-related DEmRNAs in IDD

**Figure 1:**
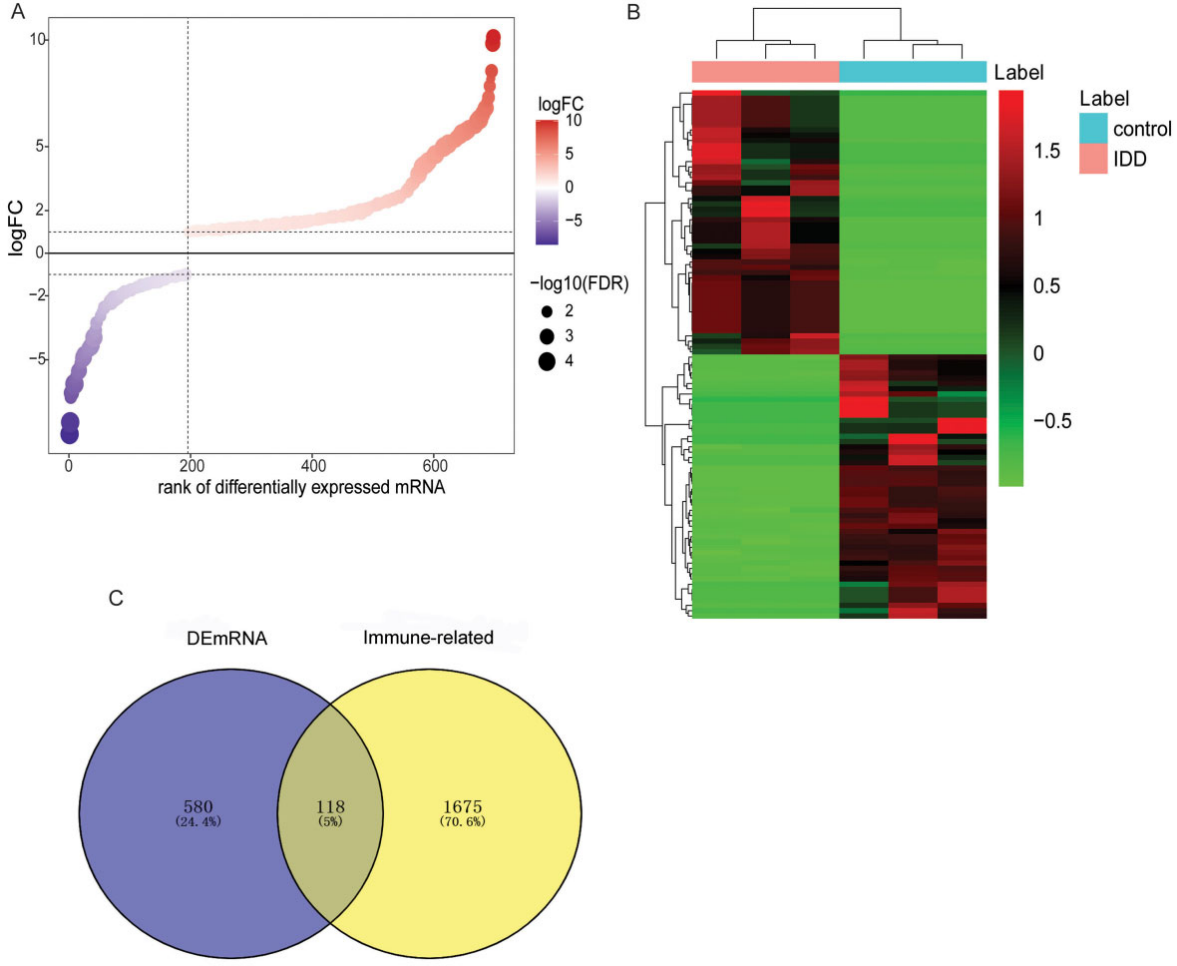
Identification of immune-related differentially expressed mRNAs (DEmRNAs) in intervertebral disc degeneration (IDD). **A**, Bubble rank map of DEmRNAs. Red and purple represent up-regulated and down-regulated RNAs, respectively. **B**, Heat map of top100 DEmRNAs. Rows and columns represent DEmRNAs and samples, respectively. The color cluster tree on the right indicates the relative expression level of mRNA. Red and green represent up-regulated and down-regulated, respectively. **C**, Venn diagram of DEmRNAs and immune-related genes.

Compared with the control group, 698 DEmRNAs were identified in the IDD group (|logFC|>1 and FDR<0.05), among which 503 were up-regulated and 195 were down-regulated. The bubble rank and heat map of DEmRNAs are shown in Figure 1A and B. A total of 1793 immune-related genes were obtained from immport database. Subsequently, the immune-related genes and DEmRNAs were intersected, and 118 immune-related DEmRNAs were obtained (Figure 1C).

### Functional enrichment analysis of immune-related DEmRNAs

**Figure 2:**
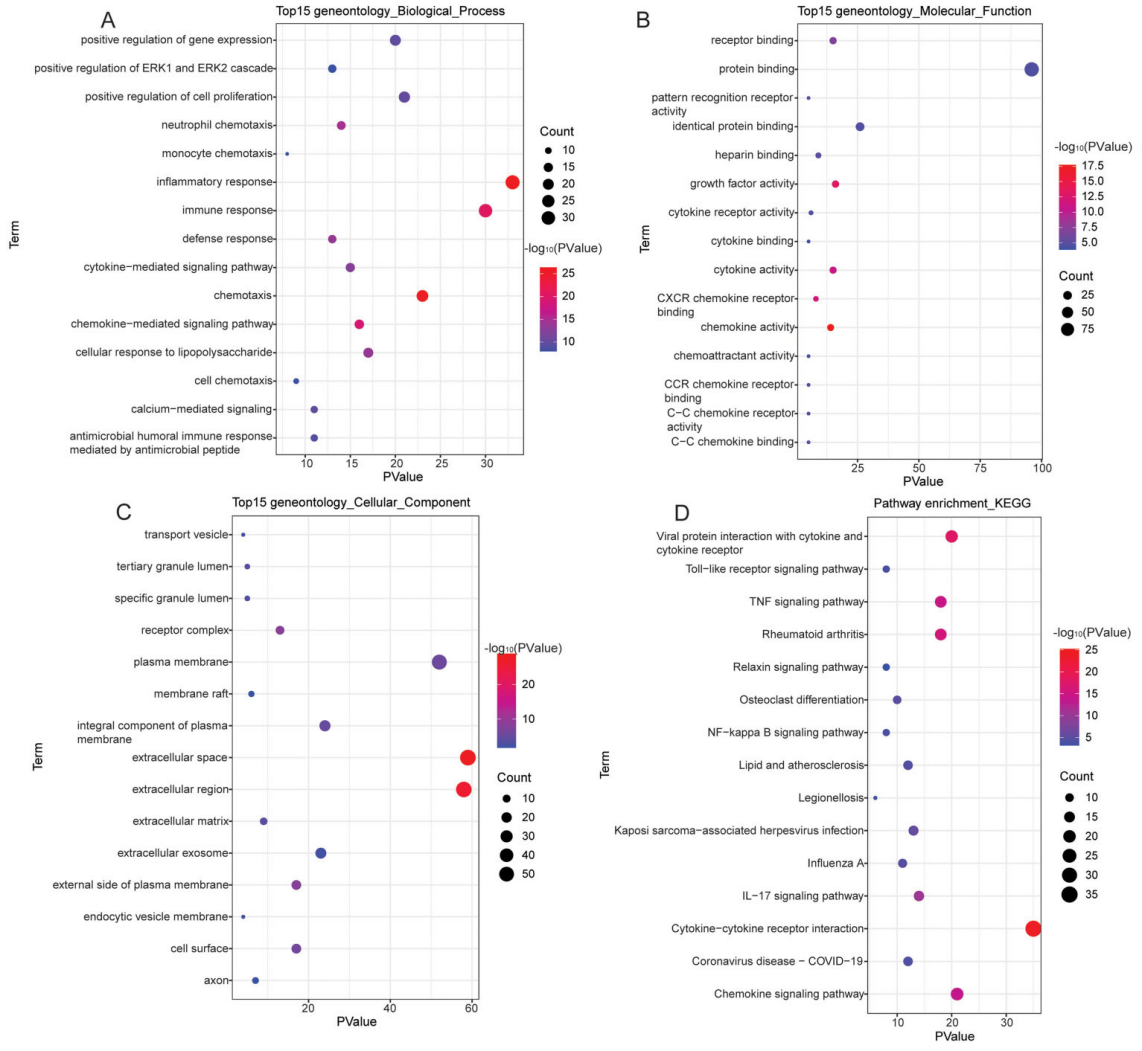
GO and KEGG functional enrichment of immune-related differentially expressed mRNAs (DEmRNAs). Top 15 significantly enriched biological processes (**A**), molecular functions (**B**), and cellular components (**C**) of immune-related DEmRNAs in GO analysis. **D**, Top 15 significantly enriched signaling pathways of immune-related DEmRNAs in KEGG analysis. The color of bubbles represents enrichment significance. The closer the color is to red, the higher the statistical significance. GO: Gene Ontology; KEGG: Kyoto Encyclopedia of Genes and Genomes.

**Figure 3:**
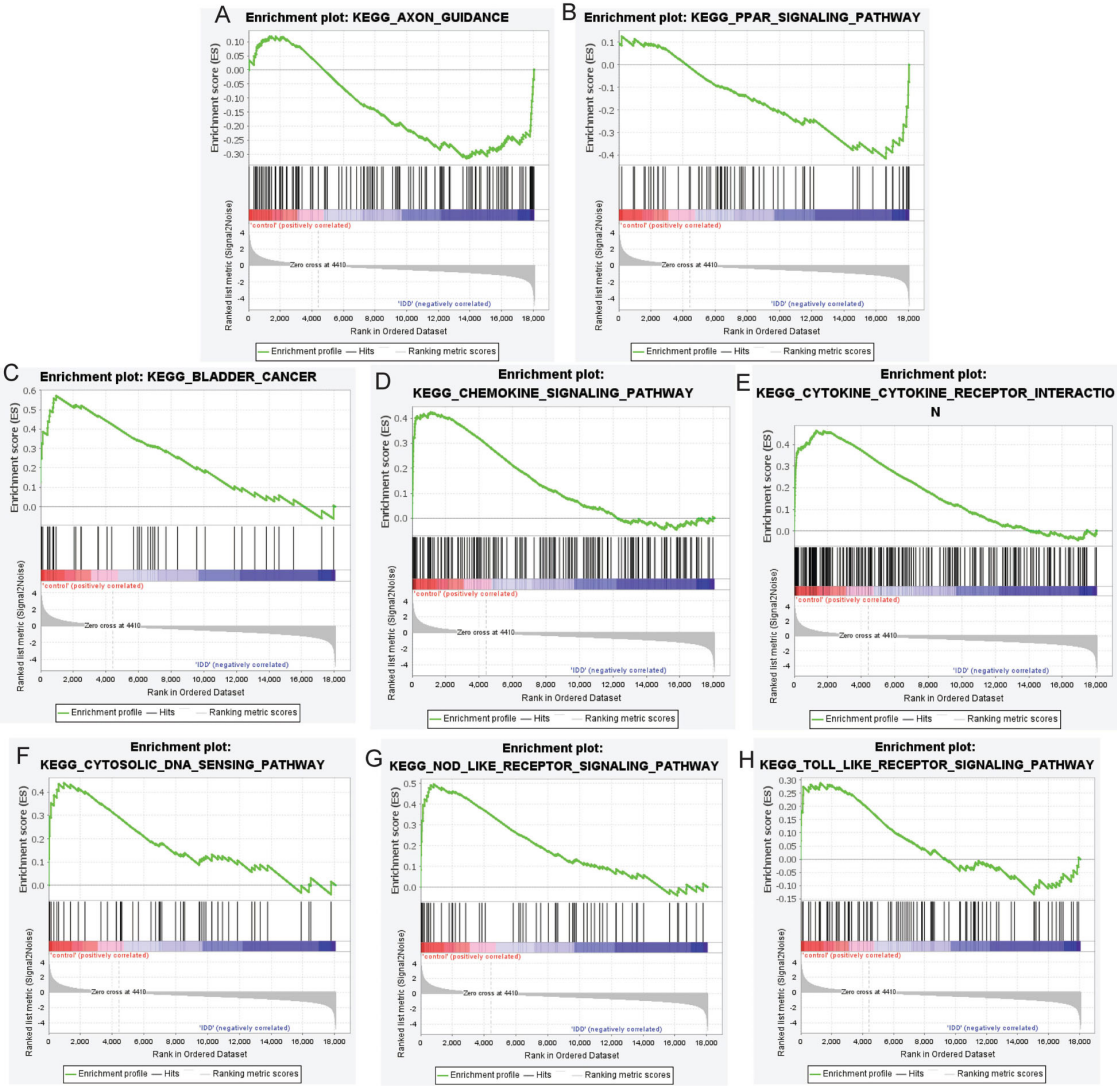
Gene set enrichment analysis (GSEA) of intervertebral disc degeneration. **A**, Axon guidance was up-regulated; **B**, PPAR signaling pathway was up-regulated; **C**, bladder cancer was down-regulated; **D**, chemokine signaling pathway was down-regulated; **E**, cytokine-cytokine receptor interaction was down-regulated; **F**, cytosolic DNA-sensing pathway was down-regulated; **G**, Nod-like receptor signaling pathway was down-regulated; **H**, GSEA toll-like receptor signaling pathway was down-regulated

GO functional enrichment analysis revealed that immune-related DEmRNAs were mainly enriched in “inflammatory response” (BP), “immune response” (BP), “protein binding” (MF), “extracellular space” (CC), and “extracellular region” (CC) (Figure 2A-C). KEGG function enrichment analysis identified 50 significantly enriched signaling pathways, such as cytokine-cytokine receptor interaction, chemokine signaling pathway, and osteoclast differentiation (Figure 2D and Supplementary Table S2). GSEA analysis showed that axon guidance and PPAR signaling pathway enriched in KEGG were up-regulated in IDD, while cytokine-cytokine receptor interaction, chemokine signaling pathway, toll-like receptor signaling pathway, Nod-like receptor signaling pathway, bladder cancer, and cytosolic DNA-sensing pathway were down-regulated in IDD (Figure 3).

### Identification of DEmiRNAs and DElncRNAs in IDD

**Figure 4:**
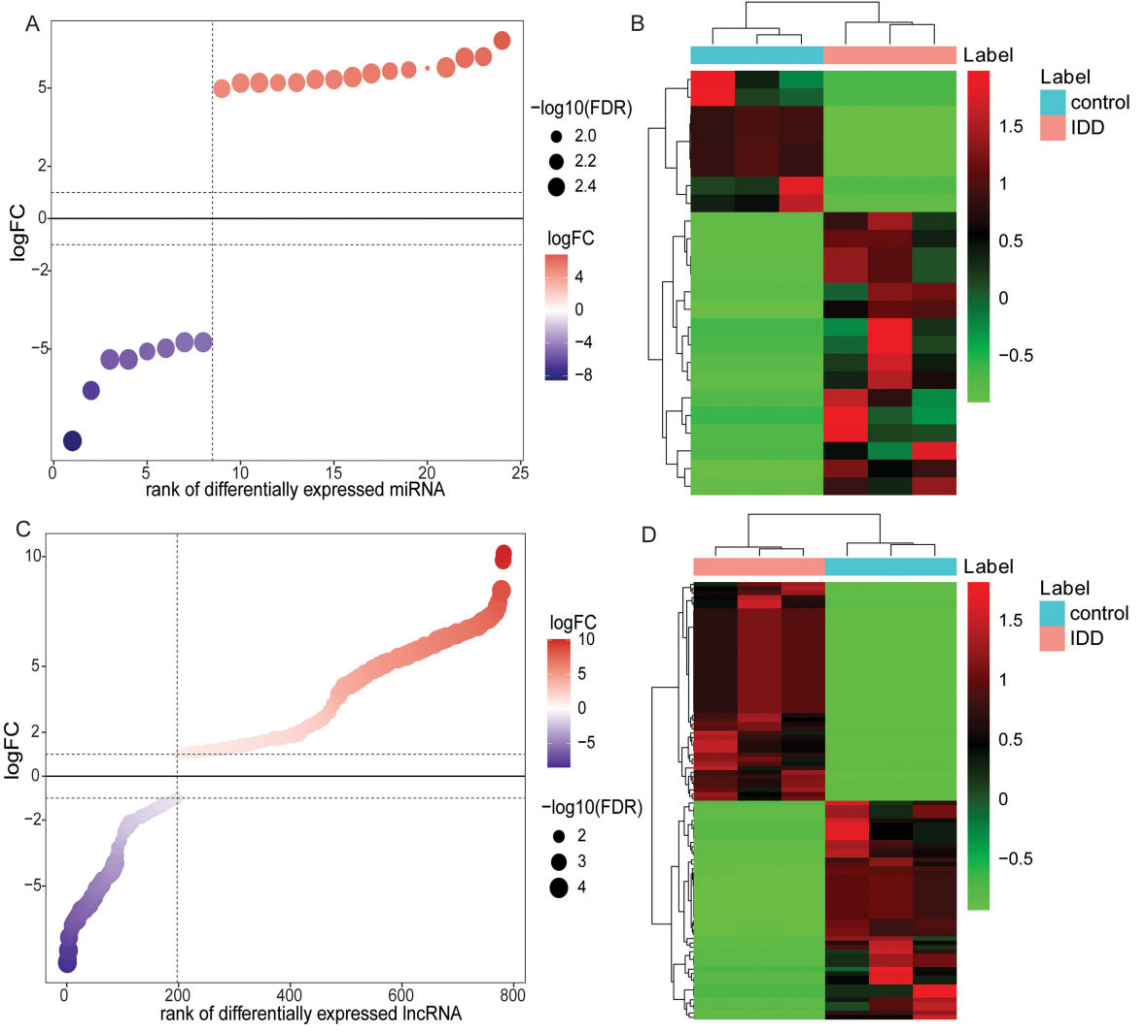
Identification of differentially expressed miRNAs (DEmiRNAs) and differentially expressed lncRNAs (DElncRNAs) in intervertebral disc degeneration (IDD). **A**, Bubble rank map of DEmiRNAs. Red and purple represent up-regulated and down-regulated lncRNAs, respectively. **B**, Heat map of DEmiRNAs. Rows and columns represent DEmiRNAs and samples, respectively. The color cluster tree on the right indicates the relative expression level of miRNA. Red and green represent up-regulated and down-regulated, respectively. **C**, Bubble rank map of DElncRNAs. Red and purple represent up-regulated and down-regulated, respectively. **D**, Heat map of top100 DElncRNAs. Rows and columns represent DElncRNAs and samples, respectively. The color cluster tree on the right indicates the relative expression level of lncRNA. Red and green represent up-regulated and down-regulated, respectively.

Compared with the control group, 24 DEmiRNAs were identified in the IDD group (|logFC|>1 and FDR<0.05), among which 16 were up-regulated and 8 were down-regulated. The bubble rank and heat map of DEmiRNAs are shown in Figure 4A and B. Compared with the control group, 782 DElncRNAs were identified in the IDD group (|logFC|>1 and FDR<0.05), among which 585 were up-regulated and 197 were down-regulated. The bubble rank and heat map of DElncRNAs are shown in Figure 4C and D.

### Construction of a ceRNA network

**Figure 5:**
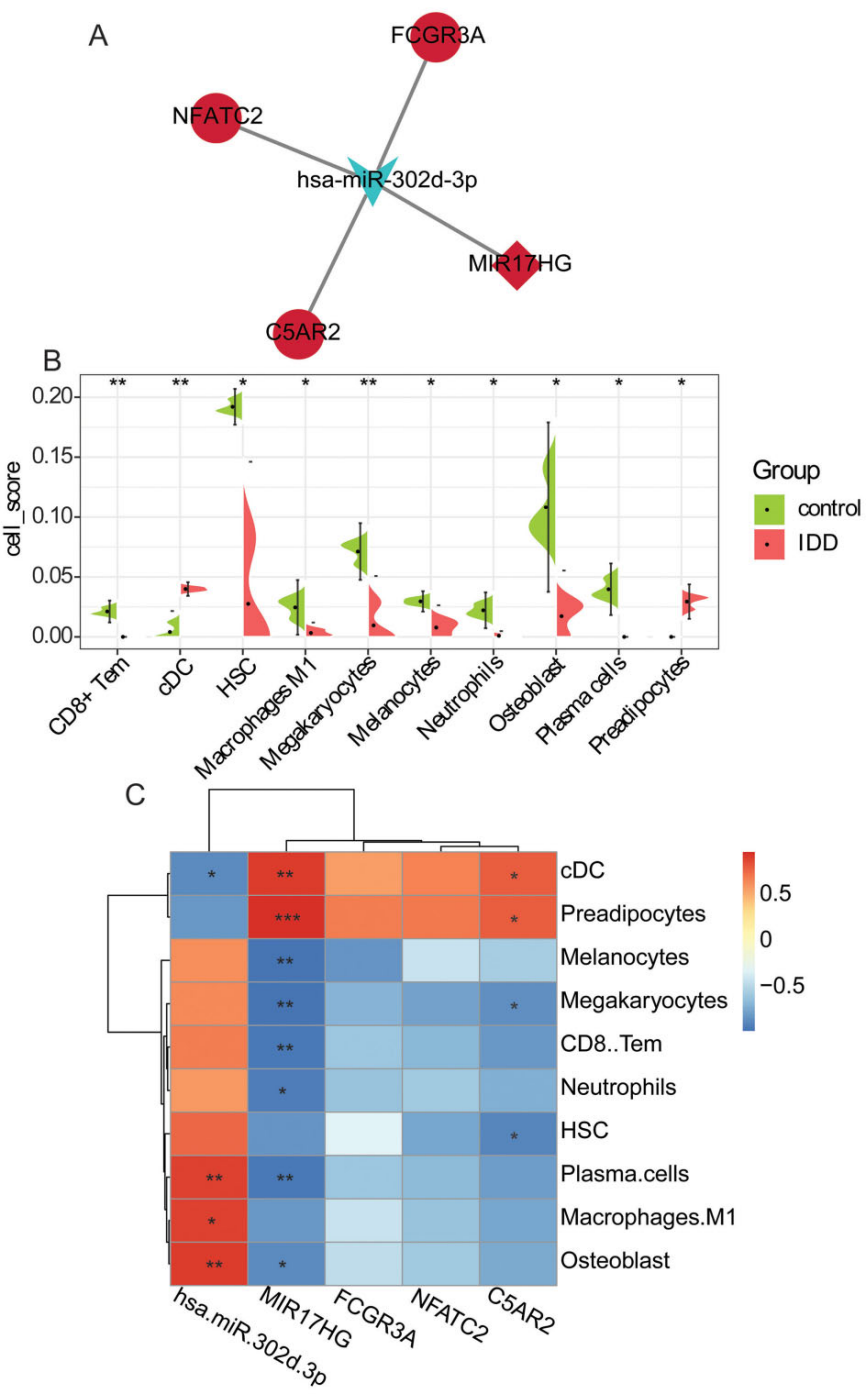
**A**, Construction of lncRNA-miRNA-mRNA (ceRNA) network. Red and blue represent up-regulated and down-regulated, respectively. Circle, rhombus, and V-shape formats represent mRNA, lncRNA, and miRNA, respectively. **B**, The distribution of 10 cell types was significantly different between normal controls and intervertebral disc degeneration (IDD) by difference analysis. **C**, Pearson correlation analysis was used to analyze the correlation between 10 cell types and complement C5a receptor 2 (C5AR2), nuclear factor of activated T cells 2 (NFATC2), Fc gamma receptor IIIa (FCGR3A), hsa-miR-302d-3p, and lncRNA miR-17-92a-1 cluster host gene (MIR17HG). Red and blue represent positive and negative correlations, respectively. cDC: conventional dendritic cell; HSC: hematopoietic stem cell. *P<0.05, **P<0.01, ***P<0.001 (*t*-test).

After the intersection treatment of the predicted miRNAs and DEmiRNAs, a pair of lncRNA-miRNA targeting relationship with negative regulation was obtained, which was hsa-miR-302d-3p-MIR17HG. In addition, 164 pairs of miRNA-mRNA targeting relationship with negative regulation were obtained after the intersection treatment of the predicted mRNAs and DEmRNAs (Supplementary Table S3). Subsequently, the lncRNA-miRNA targeting relationship pairs and miRNA-mRNA targeting relationship pairs were fused to construct a ceRNA network (Figure 5A). The ceRNA network contained 5 nodes (1 miRNA, 3 mRNAs, 1 lncRNA) and 4 edges.

### Analysis of immune cell infiltration in IDD

The XCell score was sorted into the cell infiltration matrix, and the difference analysis showed that the distribution of 10 cell types was significantly different between the normal control group and the IDD group (Figure 5B). The distributions of conventional dendritic cell (cDC) and preadipocytes in the IDD group were significantly increased, while CD8+ Tem, hematopoietic stem cell (HSC), macrophages M1, megakaryocytes, melanocytes, neutrophils, osteoblasts, and plasma cells were significantly decreased. Pearson correlation analysis was used to analyze the correlation between differential infiltration cell types and C5AR2, NFATC2, FCGR3A, hsa-miR-302d-3p, and MIR17HG (Figure 5C). The results showed that NFATC2 and FCGR3A were not significantly correlated with any cell type, while C5AR2, hsa-miR-302d-3p, and MIR17HG were significantly correlated with multiple cell types (for example: cDC, CD8+ Tem, macrophages M1, neutrophils, and plasma cells).

### ROC curve analysis

**Figure 6:**
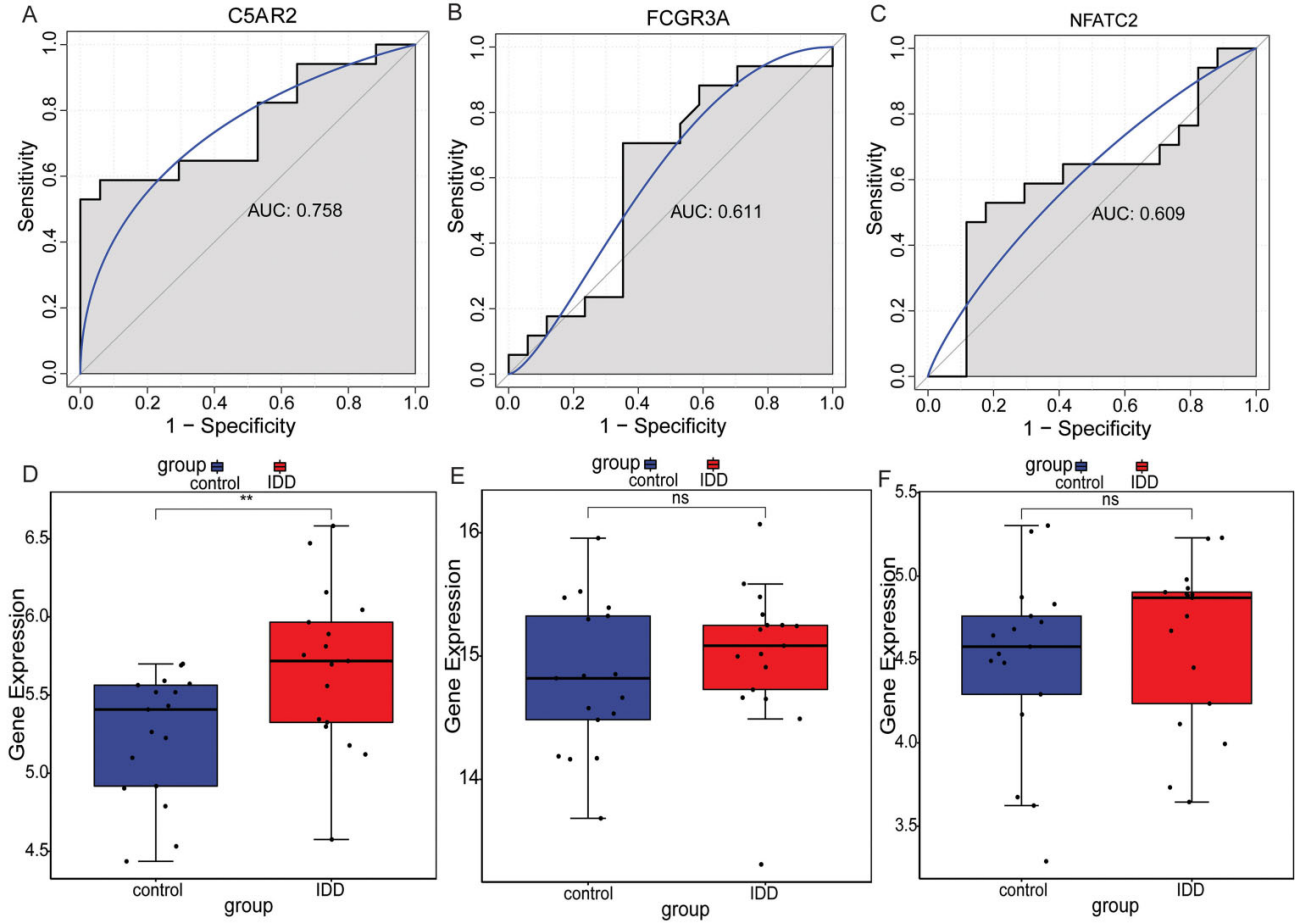
ROC analysis and expression analysis based on the GSE150408 dataset. **A**, complement C5a receptor 2 (C5AR2); **B**, Fc gamma receptor IIIa (FCGR3A); **C**, nuclear factor of activated T cells 2 (NFATC2). Electronic expression verification of C5AR2 (**D**); FCGR3A (**E**); NFATC2 (**F**). 0.6<AUC<0.7 indicates sufficient diagnostic accuracy, and AUC≥0.7 indicates good diagnostic accuracy. Data are reported as median and interquartile range. **P<0.01 (Wilcoxon test); ns: no statistical significance. ROC: receiver operator characteristic; AUC: area under the curve; IDD: intervertebral disc degeneration.

ROC analysis of C5AR2, FCGR3A, and NFATC2 was performed in the GSE150408 dataset to assess potential diagnostic value. The results showed that the AUC values of C5AR2, FCGR3A, and NFATC2 were 0.758, 0.611, and 0.609, respectively (Figure 6A-C), indicating that C5AR2 had good diagnostic accuracy, and FCGR3A and NFATC2 had sufficient diagnostic accuracy, and the three may be potential diagnostic markers of IDD. Moreover, the results of ROC analysis in the GSE167199 dataset showed that the AUC values of C5AR2, FCGR3A, and NFATC2 were all 1 (Supplementary Figure S1), which also implied their potential diagnostic value. Subsequently, expression analysis showed that C5AR2, FCGR3A, and NFATC2 were up-regulated in the IDD group (Figure 6D-F), which was consistent with the results of differential expression analysis in the GSE167199 dataset (Supplementary Figure S2).

### Real-time PCR validation of the expression levels of C5AR2, NFATC2, FCGR3A, hsa-miR-302d-3p, and MIR17HG

**Figure 7:**
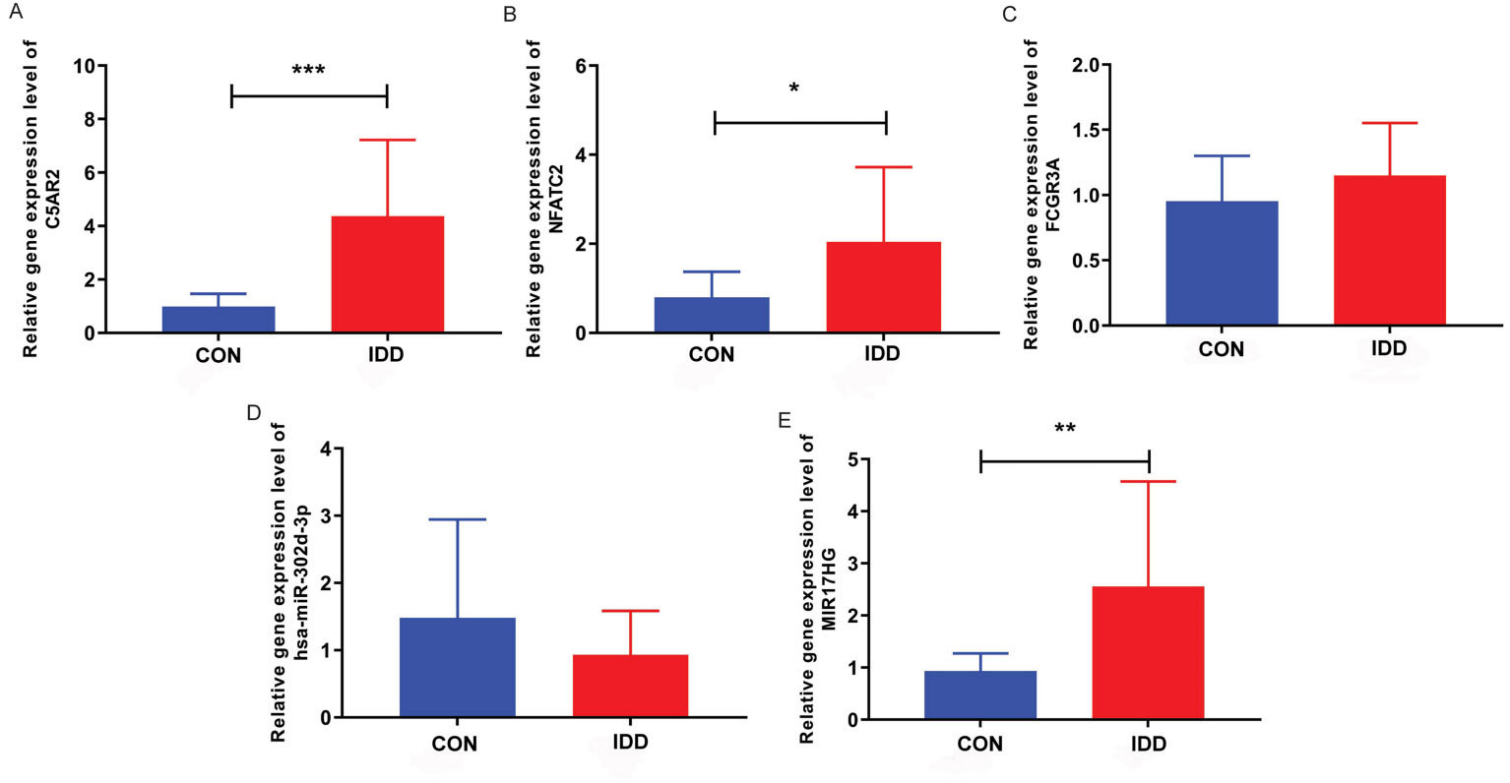
The expression levels of complement C5a receptor 2 (C5AR2) (**A**), nuclear factor of activated T cells 2 (NFATC2) (**B**), Fc gamma receptor IIIa (FCGR3A) (**C**), hsa-miR-302d-3p (**D**), and lncRNA miR-17-92a-1 cluster host gene (MIR17HG) (**E**) were verified through real-time PCR. CON: control group; IDD: intervertebral disc degeneration group. Data are reported as mean and SD. *P<0.05; **P<0.01; ***P<0.001 (*t*-test).

Real-time PCR was performed based on 12 IDD blood samples and 15 control blood samples to verify the expression of C5AR2, NFATC2, FCGR3A, hsa-miR-302d-3p, and MIR17HG. All primer sequences are shown in Supplementary Table S4. Real-time PCR results showed that C5AR2, NFATC2, FCGR3A, and MIR17HG were up-regulated in the IDD group, while hsa-miR-302d-3p was down-regulated (Figure 7). The specific 2^-△△CT^ values for C5AR2, NFATC2, FCGR3A, hsa-miR-302d-3p, and MIR17HG are shown in Supplementary Table S5. Moreover, the real-time PCR results were consistent with the results of analyses based on public datasets, which again hinted that C5AR2, NFATC2, FCGR3A, hsa-miR-302d-3p, and MIR17HG play an important regulatory role in IDD. In addition, the lack of significance of FCGR3A and hsa-miR-302d-3p may be caused by sample heterogeneity. Therefore, it is necessary to expand the sample size and conduct a series of *in vitro* experimental verifications in the future.

## Discussion

With the understanding of intervertebral disc biology, gene therapy has shown great hope in the treatment of IDD. The advantage of gene therapy in IDD is that it is not limited to the introduction of a single gene, and the combination of different genes has the potential to obtain a better synergistic therapeutic effect, which may be lasting and conducive to disc regeneration. In this study, 5 molecules (C5AR2, NFATC2, FCGR3A, hsa-miR-302d-3p, and MIR17HG) that may play a regulatory role in the immune regulation of IDD were identified by differential expression analysis and ceRNA network construction. Inducing changes in the expression of these genes through gene therapy may contribute to the treatment of diseases, but further validation is needed.

C5AR2 is a powerful regulator of innate and adaptive immunity. At present, whether C5AR2 is a proinflammatory or anti-inflammatory receptor is controversial. By regulating C5a, C5AR2, on the one hand, may limit C5a/C5AR1-mediated cell activation, thus acting as an anti-inflammatory. On the other hand, C5AR2 may induce G-protein independent activation of immune cells, thereby exerting proinflammatory effects (9). C5AR2 plays an important role in bone homeostasis, affecting the osteoblast/osteoclast balance. In addition, the expression level of C5AR2 in different immune cell populations also affects the inflammatory response in fracture healing (10). Moreover, C5AR2, as an immune related gene, is found to be highly expressed in IDD (6).

NFATC2 (also known as NFAT1) plays an important role in T cell development and function and promotes CD8+ T cell exhaustion. In NFATC2-deficient mice, the early innate inflammatory response is delayed. Moreover, NFATC2 is an important factor controlling mast cell activation and plays an important role in all aspects of mast cell-mediated immunity *in vivo* (11). NFATC2 may also mediate osteochondral differentiation (12). NFATC2 activation in osteoblasts inhibits bone formation and leads to osteopenia in cancellous bone, and NFATC2 activation also impairs osteoblast generation *in vitro* (13). The Fc gamma receptor IIIa (FCGR3A) gene encodes the FcγRIIIa receptor present in macrophages, NK, and γδT cells and has been implicated in a variety of immune-mediated diseases. Abatacept is an immunosuppressant, which is used for the treatment of rheumatoid arthritis, psoriatic arthritis, and juvenile idiopathic arthritis, and FCGR3A can affect the response to abatacept treatment in rheumatoid arthritis (14). The FCGR3A polymorphism is also associated with susceptibility to rheumatoid arthritis (15). Moreover, FCGR3A may also be involved in the regulation of disc herniation (16).

In the mouse model of acute myocardial infarction, hsa-miR-302d-3p is involved in inhibiting the inflammatory response after acute myocardial infarction (17). Hsa-miR-302d-3p may also mediate chondrocyte proliferation and apoptosis in osteoarthritis (18,19). MIR17HG can promote the occurrence and development of colorectal cancer by activating the NF-κB pathway and PD-L1-induced immune suppression (20). MIR17HG can modulate the miR-21/PTEN axis to regulate chemoresistance of acute myeloid leukemia cells (21). MIR17HG gene variant is also associated with susceptibility to femoral head necrosis (22).

In this study, we found that C5AR2, NFATC2, FCGR3A, hsa-miR-302d-3p, and MIR17HG were abnormally expressed in IDD. Combined with previous studies, we hypothesized that the C5AR2/NFATC2/FCGR3A-hsa-miR-302d-3p-MIR17HG axis may play an important mediating role in the occurrence and development of IDD. In addition, our findings suggested that C5AR2, FCGR3A, and NFATC2 may be potential diagnostic markers of IDD. However, it is crucial to note that there is a notable difference in sample sources within this study. The GSE167199 dataset utilized for analysis was derived from nucleus pulposus samples, which are integral components of the intervertebral disc. These samples are characterized by a distinct micro-environment. The real-time PCR expression validation was based on blood samples, which circulate throughout the body and are exposed to a wide range of systemic factors. This divergence in sample sources can significantly impact the representativeness and accuracy of the results. Given that the nucleus pulposus and blood have entirely different cell compositions and gene expression regulation mechanisms, the gene expression patterns of the two are likely to vary substantially. Thus, the conclusions drawn from this study are inevitably subject to certain limitations. To enhance the reliability and generalizability of the findings, future research should focus on using consistent sample sources.

DCs are divided into plasmacytoid DCs, monocyte-derived DCs, and cDCs. cDCs can effectively initiate T follicular helper cells (Tfh) and play a role in immune response (23). CD8+ Tem play an important role in systemic immune detection and protective immunity (24). Neutrophils are important immune cells in the immune system and play important regulatory roles in acute injury and repair, cancer, autoimmunity, and chronic inflammatory processes (25). The presence of neutrophils at the infection site is critical, and the depletion of neutrophils in the blood can lead to severe immune deficiency in humans (25). Moreover, neutrophils play a regulatory role in IDD (26). Plasma cells are ultimately differentiated B cells and effectors of humoral immunity. Neutrophils can secrete protective antibodies after encountering antigens, which is important for immune protection (27,28). M1 macrophages are important regulatory cells in immune regulation, which plays an important role in the progression of IDD (29,30). Therefore, we hypothesized that cDC, CD8+ Tem, M1 macrophages, neutrophils, and plasma cells may play important regulatory roles in the immune mechanism of IDD. However, specific mechanisms of the C5AR2-hsa-miR-302d-3p-MIR17HG axis in regulating related immune cells in IDD need to be further explored.

To understand the immune signaling pathways that may be involved in IDD, we also performed KEGG and GSEA enrichment analysis. Chemokine signaling plays an important role in regulating inflammatory and immune responses (31,32). Cytokine-cytokine receptor interaction and chemokine signaling pathway are important enrichment pathways and may also play a regulatory role in IDD (33,34). The toll-like receptor is a protective immune sentinel, and the disorder of TLR signal cascade will lead to many human diseases (35). Toll-like receptor 2 and toll-like receptor 4 are important target molecules in the toll-like receptor signaling pathway. Studies have shown that they play an important role in the molecular mechanism of IDD (36,37). Nod-like receptors are a family of pattern recognition receptors that mediate the innate immune system (38). Nod-like receptor protein 3 inflammasome activation is associated with IDD (39). Moreover, a previous study also showed that Nod-like receptor signaling pathway is an important signaling pathway in IDD and may play a role in IDD development (40). Based on our findings, we also hypothesized that cytokine-cytokine receptor interaction, chemokine signaling pathway, toll-like receptor signaling pathway, and Nod-like receptor signaling pathway have important roles in the immune response of IDD, and continued exploration of their potential roles is beneficial to the understanding of the immune mechanism of IDD.

However, this study also has some limitations. Firstly, the data used in this study were from the public GSE167199 dataset and from nucleus pulposus samples, while the RT-PCR used blood samples. Therefore, a large number of samples of nucleus pulposus should be collected for further study. Secondly, due to the lack of corresponding clinical information in the public GSE167199 dataset, statistics of clinical information and sample screening cannot be conducted to eliminate the influence of confounding factors. Therefore, it is necessary to record the clinical information of individuals during the collection of nucleus pulposus samples in the later stage to facilitate further research. Thirdly, the specific molecular mechanisms of the identified key immune-related molecules, cells, and signaling pathways in IDD are still unclear, so a large number of *in vitro* experiments are needed for further study.

## Conclusions

In this study, we found that the immune-related mRNAs C5AR2, FCGR3A, and NFATC2 may be potential diagnostic markers of IDD. Moreover, we also found that a large number of immune-related signaling pathways, such as cytokine-cytokine receptor interaction, chemokine signaling pathway, toll-like receptor signaling pathway, and Nod-like receptor signaling pathway, which were significantly enriched, may play a role in regulating the occurrence and progression of IDD through KEGG and GSEA enrichment analysis. Interestingly, we also found that the C5AR2-hsa-miR-302d-3p-MIR17HG axis may play a role in immune regulation in the IDD microenvironment. Exploring the role of IDD key markers in immune cell infiltration and immune microenvironment changes from the immune perspective is of great significance for revealing the pathogenesis of IDD.

## Supplementary Material

Click here to [pdf].
