# Supplementary material for: Unveiling immune mechanisms and potential biomarkers in intervertebral disc degeneration through integrated analysis
Source: Braz J Med Biol Res. 2025 Jun 20;58:e14553. doi: 10.1590/1414-431X2025e14553 (PMC12184963; doi:10.1590/1414-431X2025e14553)
Supplement: Supplementary file 1 [file 1414-431X-bjmbr-58-e14553-suppl.pdf]

Figure S1. ROC analysis of complement C5a receptor 2 (C5AR2), Fc gamma receptor IIIa (FCGR3A), and nuclear factor of activated T cells 2 (NFATC2) were performed based on GSE167199 dataset.

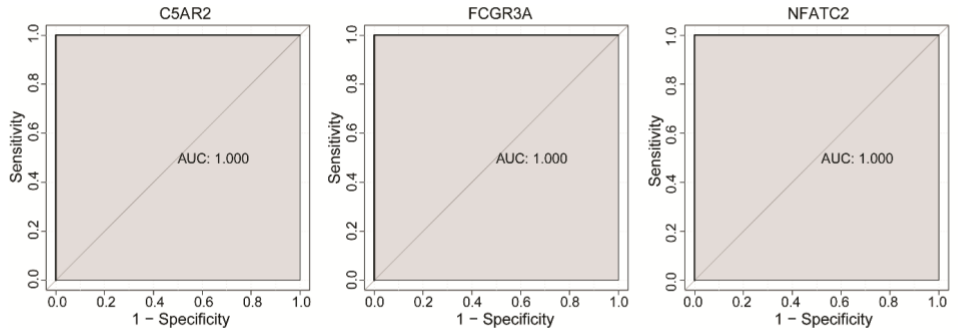

Figure S2. Differential expression boxplot of complement C5a receptor 2 (C5AR2), Fc gamma receptor IIIa (FCGR3A), and nuclear factor of activated T cells 2 (NFATC2) in the GSE167199 dataset. Data are reported as median and interquartile range. *P<0.05; **P<0.01 (Wilcoxon test). IDD: intervertebral disc degeneration.

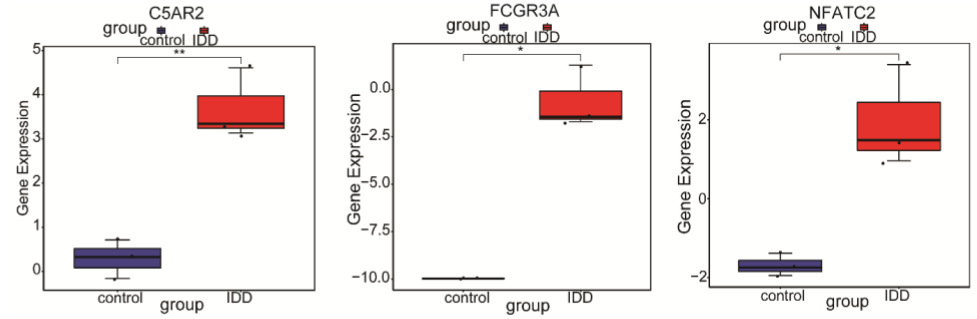

Table S1. Clinical information of individuals included in the RT-PCR validation.

Groups	Number	Gender	Age	Height (cm)	Weight (kg)	Body mass index (BMI)	Smoking history	Drinking history	Family history	Heart disease	High blood pressure	Diabetes	Lumbar spine disease
Control	1	Female	40	165	52	19.1	No	No	No	No	No	No	No
	2	Female	54	163	69	25.97	No	No	No	No	No	No	No
	3	Female	53	160	54	21.1	No	No	No	No	No	No	No
	4	Female	35	165	58	21.3	No	No	No	No	No	No	No
	5	Male	70	176	80	25.8	No	No	No	No	Yes	No	No
	6	Male	55	174	75	24.8	Yes	No	No	No	No	No	No
	7	Female	59	160	55	21.5	No	No	No	No	No	No	No
	8	Female	54	162	50	19.1	No	No	No	No	No	No	No
	9	Female	33	165	54	19.8	No	No	No	No	No	No	No
	10	Female	32	164	48	17.8	No	No	No	No	No	No	No
	11	Female	43	166	60	21.8	No	No	No	No	No	No	No
	12	Female	58	165	58	21.3	No	No	No	No	No	No	No
	13	Female	34	167	56	20.1	No	No	No	No	No	No	No
	14	Female	74	158	52	20.8	No	No	No	No	Yes	No	No
	15	Male	44	175	80	26.1	Yes	No	No	No	No	No	No
IDD	1	Female	63	162	70	26.67	No	No	No	No	Yes	No	Yes
	2	Male	32	175	92	30.04	No	No	No	No	No	No	Yes
	3	Male	54	169	80	28.01	No	No	No	No	Yes	No	Yes
	4	Female	58	158	58	23.23	No	No	No	No	No	No	Yes
	5	Female	58	158	60	24.03	No	No	No	No	No	No	Yes
	6	Male	52	180	70	21.6	Yes	No	No	No	Yes	No	Yes
	7	Female	63	150	70	31.11	No	No	No	No	Yes	No	Yes
	8	Male	45	178	71	22.4	Yes	No	No	No	No	No	Yes
	9	Female	38	155	58	24.14	No	No	No	No	No	No	Yes
	10	Female	66	158	75	30.04	No	No	No	No	Yes	No	Yes
	11	Male	40	165	90	33.05	No	No	No	No	No	No	Yes
	12	Female	73	165	50	18.36	No	No	No	No	Yes	No	Yes

IDD: intervertebral disc degeneration.

Table S2. KEGG enrichment analysis results of immune related differentially expressed mRNAs.

Term	Count	P-value	Genes
hsa04060:Cytokine-cytokine receptor interaction	35	5.83E-26	CXCL6, CXCL8, CSF1, CXCR4, CXCL1, CXCL3, CXCL2, CXCL5, CX3CL1, IL36B, CCL5, CCL2, CCR8, TNFSF11, CCR7, IL36RN, IL12RB1, IL13RA2, CCL18, PF4V1, CCR3, IL32, IL33, XCR1, CCL20, TNFSF15, IL31RA, INHBB, IFNLR1, NGF, GDF6, BMP6, CXCL10, IL6, IL7R
hsa04061:Viral protein interaction with cytokine and cytokine receptor	20	1.08E-18	CXCL6, XCR1, CXCL8, CSF1, CCL20, CXCR4, CXCL1, CXCL3, CXCL2, CXCL5, CX3CL1, CXCL10, IL6, CCL5, CCL2, CCR8, CCR7, CCL18, PF4V1, CCR3
hsa05323:Rheumatoid arthritis	18	1.68E-16	CD86, CXCL6, HLA-DRB5, FLT1, CXCL8, CSF1, CCL20, CXCL1, FOS, CXCL3, CXCL2, CXCL5, VEGFA, IL6, CCL5, CCL2, TNFSF11, TLR2
hsa04668:TNF signaling pathway	18	4.60E-15	CXCL6, CSF1, CCL20, VEGFC, TNFAIP3, CXCL1, FOS, NOD2, CXCL3, CXCL2, MMP9, CXCL5, CX3CL1, CXCL10, SOCS3, IL6, CCL5, CCL2
hsa04062:Chemokine signaling pathway	21	2.41E-14	CXCL6, XCR1, SHC3, CXCL8, CCL20, CXCR4, CXCL1, CXCL3, CXCL2, CXCL5, CX3CL1, CXCL10, HCK, CCL5, PLCG2, CCL2, CCR8, CCR7, CCL18, PF4V1, CCR3
hsa04657:IL-17 signaling pathway	14	3.30E-11	CXCL6, CXCL8, CCL20, TNFAIP3, CXCL1, FOS, CXCL3, CXCL2, MMP9, CXCL5, CXCL10, IL6, LCN2, CCL2
hsa05167:Kaposi sarcoma-associated herpesvirus infection	13	1.80E-06	CD86, CXCL8, NFATC2, CXCL1, FOS, CXCL3, CXCL2, VEGFA, HCK, IL6, PLCG2, CCR8, CCR3
hsa04380:Osteoclast differentiation	10	1.38E-05	SOCS3, FCGR3A, TYROBP, CSF1, BTK, PLCG2, NFATC2, TNFSF11, PPARG, FOS
hsa05164:Influenza A	11	2.27E-05	IL33, CXCL10, SOCS3, IL6, HLA-DRB5, CXCL8, RSAD2, CCL5, MX2, CCL2, TLR7
hsa05417:Lipid and atherosclerosis	12	3.03E-05	IL6, CXCL8, CCL5, NFATC2, CCL2, CXCL1, PPARG, FOS, CXCL3, CXCL2, MMP9, TLR2
hsa05171:Coronavirus disease - COVID-19	12	6.08E-05	CXCL10, IL6, C5, CXCL8, MX2, C5AR1, PLCG2, CCL2, TLR7, ISG15, FOS, TLR2
hsa04620:Toll-like receptor signaling pathway	8	1.73E-04	CD86, CXCL10, IL6, CXCL8, CCL5, TLR7, FOS, TLR2
hsa04064:NF-kappa B signaling pathway	8	1.73E-04	CXCL8, BTK, PLCG2, TNFSF11, TNFAIP3, CXCL1, CXCL3, CXCL2
hsa05134:Legionellosis	6	4.54E-04	IL6, CXCL8, CXCL1, CXCL3, CXCL2, TLR2
hsa04926:Relaxin signaling pathway	8	6.45E-04	SHC3, VEGFC, FOS, NOS1, RLN1, RXFP1, MMP9, VEGFA
hsa04621:NOD-like receptor signaling pathway	9	0.001144	IL6, CXCL8, CCL5, TNFAIP3, CCL2, CXCL1, NOD2, CXCL3, CXCL2
hsa05120:Epithelial cell signaling in Helicobacter pylori infection	6	0.001167	CXCL8, CCL5, PLCG2, CXCL1, CXCL3, CXCL2
hsa04014:Ras signaling pathway	10	0.001376	FGF5, FLT1, SHC3, CSF1, PLCG2, VEGFC, NGF, FGFR3, FGFR2, VEGFA
hsa04020:Calcium signaling pathway	10	0.001593	FGF5, FLT1, PLCG2, VEGFC, CXCR4, NOS1, NGF, FGFR3, FGFR2, VEGFA
hsa05133:Pertussis	6	0.001689	CXCL6, IL6, C5, CXCL8, FOS, CXCL5
hsa01521:EGFR tyrosine kinase inhibitor resistance	6	0.002006	IL6, SHC3, PLCG2, FGFR3, FGFR2, VEGFA

hsa04662:B cell receptor signaling pathway	6	0.002365	INPP5D, BTK, PLCG2, NFATC2, PTPN6, FOS
hsa05200:Pathways in cancer	15	0.0026	CXCL8, WNT5A, VEGFC, CXCR4, FOS, MMP9, VEGFA, FGF5, IL6, PLCG2, PPARG, IL12RB1, IL7R, FGFR3, FGFR2
hsa05163:Human cytomegalovirus infection	9	0.004025	IL6, CXCL8, CCL5, NFATC2, CCL2, CXCR4, CCR3, CX3CL1, VEGFA
hsa04360:Axon guidance	8	0.00463	ROBO2, SEMA6A, SEMA3D, WNT5A, PLXNA2, PLCG2, NFATC2, CXCR4
hsa04936:Alcoholic liver disease	7	0.005524	IL6, C5, CXCL8, C5AR1, CXCL1, CXCL3, CXCL2
hsa04933:AGE-RAGE signaling pathway in diabetic complications	6	0.005559	IL6, CXCL8, PLCG2, VEGFC, CCL2, VEGFA
hsa05142:Chagas disease	6	0.006042	IL6, CXCL8, CCL5, CCL2, FOS, TLR2
hsa05146:Amoebiasis	6	0.006042	IL6, CXCL8, CXCL1, CXCL3, CXCL2, TLR2
hsa05321:Inflammatory bowel disease	5	0.006312	IL6, HLA-DRB5, NOD2, IL12RB1, TLR2
hsa04151:PI3K-Akt signaling pathway	11	0.006839	FGF5, IL6, FLT1, CSF1, VEGFC, NGF, IL7R, FGFR3, FGFR2, TLR2, VEGFA
hsa05169:Epstein-Barr virus infection	8	0.008093	CXCL10, IL6, HLA-DRB5, BTK, PLCG2, TNFAIP3, ISG15, TLR2
hsa04015:Rap1 signaling pathway	8	0.009912	FGF5, FLT1, CSF1, VEGFC, NGF, FGFR3, FGFR2, VEGFA
hsa04630:JAK-STAT signaling pathway	7	0.010303	SOCS3, IL6, PTPN6, IFNLR1, IL7R, IL12RB1, IL13RA2
hsa03320:PPAR signaling pathway	5	0.010397	FABP3, FABP4, FABP5, PPARG, ANGPTL4
hsa05219:Bladder cancer	4	0.011189	CXCL8, FGFR3, MMP9, VEGFA
hsa05140:Leishmaniasis	5	0.011379	FCGR3A, HLA-DRB5, PTPN6, FOS, TLR2
hsa04935:Growth hormone synthesis, secretion and action	6	0.011396	SOCS3, SHC3, PLCG2, FOS, SSTR1, SSTR5
hsa04650:Natural killer cell mediated cytotoxicity	6	0.014325	FCGR3A, TYROBP, SHC3, PLCG2, NFATC2, PTPN6
hsa04672:Intestinal immune network for IgA production	4	0.018119	CD86, IL6, HLA-DRB5, CXCR4
hsa04010:MAPK signaling pathway	9	0.018651	FGF5, FLT1, CSF1, VEGFC, FOS, NGF, FGFR3, FGFR2, VEGFA
hsa05144:Malaria	4	0.019119	IL6, CXCL8, CCL2, TLR2
hsa05162:Measles	6	0.021031	IL6, MX2, TNFAIP3, TLR7, FOS, TLR2
hsa04613:Neutrophil extracellular trap formation	7	0.021136	FCGR3A, C5, C5AR1, PLCG2, TLR7, FPR2, TLR2
hsa04080:Neuroactive ligand-receptor interaction	10	0.021832	C5, MTNR1A, C5AR1, S1PR1, PTH1R, FPR2, RLN1, SSTR1, RXFP1, SSTR5
hsa05150:Staphylococcus aureus infection	5	0.023743	FCGR3A, HLA-DRB5, C5, C5AR1, FPR2
hsa04659:Th17 cell differentiation	5	0.034586	IL6, HLA-DRB5, NFATC2, FOS, IL12RB1
hsa04623:Cytosolic DNA-sensing pathway	4	0.034868	IL33, CXCL10, IL6, CCL5
hsa05161:Hepatitis B	6	0.037347	IL6, CXCL8, NFATC2, FOS, MMP9, TLR2
hsa04917:Prolactin signaling pathway	4	0.04543	SOCS3, SHC3, TNFSF11, FOS

Table S3. Pairs (n=164) of miRNA-mRNA targeting relationship with negative regulation were obtained after the intersection treatment of the predicted mRNAs and differentially expressed mRNAs.

Number	miRNA	mRNA	miRNA (up/down)	mRNA (up/down)
1	hsa-miR-1266-5p	RXFP1	up	down
2	hsa-miR-1266-5p	IL33	up	down
3	hsa-miR-1266-5p	IL12RB1	up	down
4	hsa-miR-1266-5p	NOD2	up	down
5	hsa-miR-1266-5p	MX2	up	down
6	hsa-miR-1266-5p	CXCL6	up	down
7	hsa-miR-1266-5p	STC1	up	down
8	hsa-miR-1266-5p	VEGFA	up	down
9	hsa-miR-302d-3p	C5AR2	down	up
10	hsa-miR-302d-3p	NFATC2	down	up
11	hsa-miR-302d-3p	FCGR3A	down	up
12	hsa-miR-3131	NGF	up	down
13	hsa-miR-3131	SHC3	up	down
14	hsa-miR-3131	IL12RB1	up	down
15	hsa-miR-3131	CSF1	up	down
16	hsa-miR-3131	IL36RN	up	down
17	hsa-miR-3131	IL7R	up	down
18	hsa-miR-3131	MX2	up	down
19	hsa-miR-3131	TNFAIP3	up	down
20	hsa-miR-3131	CXCL8	up	down
21	hsa-miR-3131	RXFP1	up	down
22	hsa-miR-3150a-3p	IL33	up	down
23	hsa-miR-3150a-3p	CSF1	up	down
24	hsa-miR-3150a-3p	INHBB	up	down

25	hsa-miR-3150a-3p	CXCL5	up	down
26	hsa-miR-3150a-3p	WNT5A	up	down
27	hsa-miR-3150a-3p	CXCL8	up	down
28	hsa-miR-3202	VEGFA	up	down
29	hsa-miR-3202	FGF5	up	down
30	hsa-miR-3202	CSF1	up	down
31	hsa-miR-3202	OASL	up	down
32	hsa-miR-3202	IL7R	up	down
33	hsa-miR-3202	CXCL5	up	down
34	hsa-miR-3202	CX3CL1	up	down
35	hsa-miR-3202	STC2	up	down
36	hsa-miR-3202	WNT5A	up	down
37	hsa-miR-3529-5p	STC2	up	down
38	hsa-miR-3529-5p	CCL5	up	down
39	hsa-miR-3529-5p	CCR7	up	down
40	hsa-miR-3529-5p	SHC3	up	down
41	hsa-miR-3529-5p	CXCL8	up	down
42	hsa-miR-3622a-3p	CCL5	up	down
43	hsa-miR-3622a-3p	RSAD2	up	down
44	hsa-miR-3622a-3p	TNFSF15	up	down
45	hsa-miR-3622a-3p	ANGPTL4	up	down
46	hsa-miR-4498	CCR7	up	down
47	hsa-miR-4498	FGF5	up	down
48	hsa-miR-4498	RSAD2	up	down
49	hsa-miR-4498	IL7R	up	down
50	hsa-miR-4498	SHC3	up	down
51	hsa-miR-4498	RXFP1	up	down
52	hsa-miR-4498	STC1	up	down
53	hsa-miR-4498	TNFSF15	up	down
54	hsa-miR-4498	CXCL10	up	down
55	hsa-miR-4498	GDF6	up	down
56	hsa-miR-4670-5p	TNFAIP3	up	down
57	hsa-miR-4738-3p	CCL5	up	down
58	hsa-miR-4738-3p	IL7R	up	down
59	hsa-miR-4738-3p	SHC3	up	down
60	hsa-miR-4738-3p	RXFP1	up	down
61	hsa-miR-4738-3p	CXCL5	up	down
62	hsa-miR-4738-3p	TNFSF15	up	down
63	hsa-miR-4738-3p	DKK1	up	down
64	hsa-miR-4738-3p	IL33	up	down
65	hsa-miR-5187-3p	CCR7	up	down
66	hsa-miR-5187-3p	FGF5	up	down
67	hsa-miR-5187-3p	LTBP1	up	down
68	hsa-miR-5187-3p	STC1	up	down
69	hsa-miR-5187-3p	GDF6	up	down
70	hsa-miR-522-3p	SOCS3	down	up
71	hsa-miR-522-3p	CD86	down	up
72	hsa-miR-5584-3p	PLCG2	down	up
73	hsa-miR-5584-3p	TGFBR3	down	up
74	hsa-miR-5584-3p	C5AR2	down	up
75	hsa-miR-5584-3p	BTK	down	up
76	hsa-miR-5584-3p	TNFSF11	down	up
77	hsa-miR-5584-3p	COLEC12	down	up
78	hsa-miR-5584-3p	IL31RA	down	up
79	hsa-miR-5584-3p	IFNLR1	down	up
80	hsa-miR-5584-3p	XCR1	down	up
81	hsa-miR-5584-3p	TDGF1	down	up
82	hsa-miR-5584-3p	S1PR1	down	up
83	hsa-miR-5584-3p	BMP6	down	up
84	hsa-miR-5584-3p	FLT1	down	up
85	hsa-miR-5584-3p	CD86	down	up
86	hsa-miR-569	SEMA3D	down	up
87	hsa-miR-569	NOS1	down	up
88	hsa-miR-5708	IL33	up	down
89	hsa-miR-5708	RSAD2	up	down
90	hsa-miR-5708	IL7R	up	down
91	hsa-miR-5787	CCR7	up	down
92	hsa-miR-5787	IL32	up	down
93	hsa-miR-5787	IL33	up	down
94	hsa-miR-5787	FGF5	up	down
95	hsa-miR-5787	RSAD2	up	down
96	hsa-miR-5787	NGF	up	down
97	hsa-miR-5787	HCK	up	down
98	hsa-miR-5787	CXCL5	up	down
99	hsa-miR-5787	TNFSF15	up	down
100	hsa-miR-5787	CXCL8	up	down

101	hsa-miR-5787	RXFP1	up	down
102	hsa-miR-6785-5p	IL36B	up	down
103	hsa-miR-6785-5p	NOD2	up	down
104	hsa-miR-6785-5p	TNFAIP3	up	down
105	hsa-miR-6785-5p	CCR7	up	down
106	hsa-miR-6785-5p	IL32	up	down
107	hsa-miR-6785-5p	IL33	up	down
108	hsa-miR-6785-5p	FGF5	up	down
109	hsa-miR-6785-5p	IL12RB1	up	down
110	hsa-miR-6785-5p	CSF1	up	down
111	hsa-miR-6785-5p	IL36RN	up	down
112	hsa-miR-6785-5p	SHC3	up	down
113	hsa-miR-6785-5p	INHBB	up	down
114	hsa-miR-6785-5p	STC1	up	down
115	hsa-miR-6785-5p	VEGFA	up	down
116	hsa-miR-6785-5p	TNFSF15	up	down
117	hsa-miR-6785-5p	CXCL10	up	down
118	hsa-miR-6785-5p	GDF6	up	down
119	hsa-miR-6785-5p	WNT5A	up	down
120	hsa-miR-6809-5p	TNFAIP3	up	down
121	hsa-miR-6809-5p	FGF5	up	down
122	hsa-miR-6809-5p	MX2	up	down
123	hsa-miR-6809-5p	SHC3	up	down
124	hsa-miR-6809-5p	FPR2	up	down
125	hsa-miR-6815-3p	CD86	down	up
126	hsa-miR-6815-3p	BTK	down	up
127	hsa-miR-6815-3p	IL31RA	down	up
128	hsa-miR-6815-3p	IFNLR1	down	up
129	hsa-miR-6815-3p	S100B	down	up
130	hsa-miR-6815-3p	PLCG2	down	up
131	hsa-miR-6815-3p	CCL18	down	up
132	hsa-miR-6815-3p	C5AR1	down	up
133	hsa-miR-6815-3p	NOS1	down	up
134	hsa-miR-6815-3p	XCR1	down	up
135	hsa-miR-6815-3p	FLT1	down	up
136	hsa-miR-6868-3p	CSF1	up	down
137	hsa-miR-6868-3p	IL7R	up	down
138	hsa-miR-6868-3p	INHBB	up	down
139	hsa-miR-6868-3p	CX3CL1	up	down
140	hsa-miR-6868-3p	WNT5A	up	down
141	hsa-miR-6868-3p	GDF6	up	down
142	hsa-miR-6880-3p	MMP9	down	up
143	hsa-miR-6880-3p	NOS1	down	up
144	hsa-miR-6880-3p	CD86	down	up
145	hsa-miR-6880-3p	BTK	down	up
146	hsa-miR-6880-3p	IL31RA	down	up
147	hsa-miR-6880-3p	XCR1	down	up
148	hsa-miR-6880-3p	PLXNA2	down	up
149	hsa-miR-6880-3p	FLT1	down	up
150	hsa-miR-6880-3p	PLCG2	down	up
151	hsa-miR-6880-3p	FGFR2	down	up
152	hsa-miR-6882-3p	IL31RA	down	up
153	hsa-miR-6882-3p	CD86	down	up
154	hsa-miR-6882-3p	DUOX1	down	up
155	hsa-miR-6882-3p	CCR3	down	up
156	hsa-miR-6882-3p	PLCG2	down	up
157	hsa-miR-6882-3p	CCL18	down	up
158	hsa-miR-6882-3p	C5AR1	down	up
159	hsa-miR-6882-3p	FCGR3A	down	up
160	hsa-miR-6882-3p	NOS1	down	up
161	hsa-miR-6882-3p	FGFR2	down	up
162	hsa-miR-9500	SEMA6A	down	up
163	hsa-miR-9500	FOS	down	up
164	hsa-miR-9500	IL31RA	down	up

Table S4. All primer sequences used for real-time PCR.

Primer name	Primer sequence (5' to 3')
GAPDH-F (internal reference)	5-GGAGCGAGATCCCTCCAAAAT-3
GAPDH-R (internal reference)	5-GGCTGTTGTCATACTTCTCATGG-3
ACTB-F (internal reference)	5-CATGTACGTTGCTATCCAGGC-3
ACTB-R (internal reference)	5-CTCCTTAATGTCACGCACGAT-3

C5AR2-F	5-GAAGAGACGACACCAGGAGC-3
C5AR2-R	5-AGGAAGATGGCGGCATACAG-3
NFATC2-F	5-CGATTCGGAGAGCCGGATAG-3
NFATC2-R	5-TGGGACGGAGTGATCTCGAT-3
FCGR3A-F	5-TGCTTTCTTGGCCAGGGTAG-3
FCGR3A-R	5-TGTCTTCTCCATCCCCACCT-3
MIR17HG-F	5-GACTCCTGACAAAATGCAGCC-3
MIR17HG-R	5-CTGTGCAGATTGAGCTCTCCT-3
hsa-U6 (internal reference)	
hsa-miR-302d-3p-F	5-TAAGTGCTTCCATGTTTGAGTGT-3

C5AR2: complement C5a receptor 2; NFATC2: nuclear factor of activated T cells 2; FCGR3A: Fc gamma receptor IIIa; MIR17HG: lncRNA miR-17-92a-1 cluster host gene. Hsa-U6 primers and hsa-miR-302d-3p reverse primer were obtained from the miRNA first strand cDNA synthesis kit (Sangon Biotech).

Table S5. Specific $2^{-\triangle\triangle CT}$ values for C5AR2, NFATC2, FCGR3A, hsa-miR-302d-3p, and MIR17HG.

Number	C5AR2	NFATC2	FCGR3A	MIR17HG	hsa-miR-302d-3p
IDD1	6.58592308	2.63057734	1.59731794	5.22209657	0.62943597
IDD2	3.60124041	2.06263381	0.97586924	1.50566838	1.55703156
IDD3	8.63485282	1.23071672	1.01849397	1.78025232	2.02623034
IDD4	8.55034685	6.41734591	1.47656902	2.56310590	1.88182376
IDD5	2.08160154	0.46879239	1.44455631	1.00667924	0.71143221
IDD6	7.20652074	0.66678646	1.06110598	6.50348513	0.50655759
IDD7	0.66185111	0.65697289	0.62021677	0.17271781	1.20759068
IDD8	5.29002916	1.79328254	0.53324663	0.23681830	0.40485154
IDD9	4.04927824	3.60581468	0.85496106	2.04142267	0.11925582
IDD10	1.14948078	0.59473112	1.51983515	1.35306000	0.08297371
IDD11	1.52065494	2.18457600	1.77476171	4.35680225	0.73143329
IDD12	3.11047854	2.18322934	0.93739937	3.92414956	1.30930226

C5AR2: complement C5a receptor 2; NFATC2: nuclear factor of activated T cells 2; FCGR3A: Fc gamma receptor IIIa; MIR17HG: lncRNA miR-17-92a-1 cluster host gene. $2^{-\triangle\triangle CT} > 1$ represents up-regulated; $2^{-\triangle\triangle CT} < 1$ represents down-regulated.
